# Intermittent alternating magnetic fields diminish metal-associated biofilm in vivo

**DOI:** 10.1038/s41598-023-49660-7

**Published:** 2023-12-17

**Authors:** Sumbul Shaikh, Norman A. Lapin, Bibin Prasad, Carolyn R. Sturge, Christine Pybus, Reed Pifer, Qi Wang, Bret M. Evers, Rajiv Chopra, David E. Greenberg

**Affiliations:** 1 Texas Prostate, Addison, TX USA; 2 Department of Radiology, University of Texas Southwestern Medical School, Dallas, TX USA; 3 Advanced Imaging Research Center, University of Texas Southwestern Medical School, Dallas, TX USA; 4 Solenic Medical, Addison, TX USA; 5 Department of Internal Medicine, Infectious Diseases and Geographic Medicine, University of Texas Southwestern Medical School, Dallas, TX USA; 6 Department of Pathology, University of Texas Southwestern Medical School, Dallas, TX USA; 7 Department of Microbiology, University of Texas Southwestern Medical School, Dallas, TX USA

**Keywords:** Applied microbiology, Biofilms, Microbiology, Biophysics, Bioenergetics

## Abstract

Prosthetic joint infection (PJI) is a complication of arthroplasty that results in significant morbidity. The presence of biofilm makes treatment difficult, and removal of the prosthesis is frequently required. We have developed a non-invasive approach for biofilm eradication from metal implants using intermittent alternating magnetic fields (iAMF) to generate targeted heating at the implant surface. The goal of this study was to determine whether iAMF demonstrated efficacy in an in vivo implant biofilm infection model. iAMF combined with antibiotics led to enhanced reduction of biofilm on metallic implants in vivo compared to antibiotics or untreated control. iAMF-antibiotic combinations resulted in a > 1 − log further reduction in biofilm burden compared to antibiotics or iAMF alone. This combination effect was seen in both *S. aureus* and *P. aeruginosa* and seen with multiple antibiotics used to treat infections with these pathogens. In addition, efficacy was temperature dependent with increasing temperatures resulting in a greater reduction of biofilm. Tissue damage was limited (< 1 mm from implant-tissue interface). This non-invasive approach to eradicating biofilm could serve as a new paradigm in treating PJI.

## Introduction

Millions of orthopedic devices are surgically implanted in patients each year to treat a wide variety of conditions including skeletal trauma, joint injuries, and osteoarthritis^1^. Frequently, implants including prosthetic joints, bone fixation hardware, and dental implants are composed of metal^2^. Amongst the most serious complications arising from the implantation of these metal prostheses is bacterial infection, occurring in 1–3% of implanted prosthetic joints and up to 30% of orthopedic trauma implants^3–5^.

For prosthetic joints, if antibiotic treatment alone fails to eliminate the infection, the gold standard for treatment of implant infections is a 2-stage surgical revision procedure^6^. While both 1-stage and 2-stage surgical revisions are common, 2-stage revision, the more onerous of the two, may be selected over 1-stage revision based on patient- and condition-specific factors^7^. However, the question of which of the two procedures is more likely to produce the better outcome remains unclear in some studies^8^. Despite these invasive procedures, relapse can be frequent^9–11^. Health care costs for treatment of prosthetic joint infections (PJI) in the US alone were approximately $1.6 billion in 2020^12^. Aside from the economic cost, the burden to patients of a 2-stage revision procedure is extremely high, resulting in many months of lost productivity and reduced quality of life. For these reasons, novel noninvasive approaches capable of treating implant infections would significantly improve management of PJI.

A major reason that antibiotic treatment of metal implant infections, such as PJI, is ineffective is due to the formation of biofilm on the implant surface^13^. Biofilm is a thin (tens to hundreds of micrometers) aggregate of bacteria and extracellular polymeric substances (EPS)^14^. EPS is generated by bacteria and forms a barrier to the surrounding environment, rendering these organisms up to a thousand-fold more resistant to antibiotics as well as providing protection from the immune system^15^. Importantly, increasing antibiotic resistance only further complicates this problem. Aside from PJI, biofilm also plays important roles in the infection of other widely used medical implants, including catheters, mechanical heart valves, and bone fixation hardware^2,16,17^. *Staphylococcus aureus* is the major pathogen associated with prosthetic joint infections (PJI)^18^. Amongst Gram-negative pathogens, *Pseudomonas aeruginosa* is a significant cause of PJI as well^19,20^.

Elimination of biofilm through non-surgical and noninvasive means is currently under development for treatment of metal implant infections broadly. Physical approaches that have been investigated to eliminate biofilm include ultrasound^21^, shock waves^22^, electrical current^23–25^ and heat-based^26–28^ methods. However, these methods are either difficult to implement in vivo or are limited in their application to metal implants. We have been developing a safer, non-invasive and potentially more effective method of biofilm removal from metal implants utilizing alternating magnetic fields (AMF)^29,30^. Delivered from outside the body, AMF is not limited by penetration depth, avoiding attenuation and distortion through biological tissue due to its long wavelength. Metal implants embedded deep within tissues are thereby exposed to AMF directly, which induces electrical currents at the implant surface, resulting in heat generation directly at the surface of the implant, where biofilm resides. Previous studies have demonstrated the feasibility and effectiveness of AMF treatment of biofilm in vitro^27,29^, resulting in a significant reduction in biofilm after only a few minutes of exposure^29,30^.

To reduce the risk of tissue damage from AMF treatment, while maintaining efficacy of biofilm eradication, brief, intermittent AMF exposures (iAMF) can be applied sequentially, elevating implant temperatures followed by a cool down period between exposures thus limiting thermal dose accumulation in surrounding tissue. When combined with antibiotics, iAMF has been found to be more effective than AMF or antibiotics alone in reducing biofilm and preventing its recurrence for up to 24 h post treatment^28,31,32^. In the present study, we employ iAMF in concert with antibiotics in mice to determine whether this approach can be effective in vivo while maintaining minimal tissue damage.

## Materials and methods

### Biofilm incubation on stainless steel beads

Biofilm was grown on stainless steel beads (6mm) using *Staphylococcus aureus* U1 (Lux UAMS-1; MSSA; generously provided by Dr. Mark Smeltzer) or *Pseudomonas aeruginosa* (Lux-PAO1, ATCC, Manassas, VA). An isolated colony was inoculated into 3 ml of Mueller–Hinton broth (MHII; Fisher Scientific), and incubated overnight at 37 °C, at 220 RPM. A bacterial suspension of 1 × 10^6^ CFU/ml was created from this culture. Biofilm was produced by placing a 6mm stainless steel bead in a conical tube containing 4 ml of the bacterial suspension and incubated in a shaking incubator at 110 RPM for 2 h at 37 °C. The initial log_10_CFU bacterial count on the un-implanted balls for *S. aureus* and *P. aeruginosa* were on average 5.0 and 4.6, respectively.

### Animal experiments

A series of animal experiments were performed to evaluate bacterial reduction with combinational therapy. The studies were approved by the Institutional Animal Care and Use Committee (IACUC) at University of Texas Southwestern Medical Center. The study is reported in accordance with ARRIVE guidelines. In addition, all experiments were performed in accordance with relevant guidelines and regulations.

### Steel ball implantation in mice

For each experimental group, approximately 8–12 mice (female 14–16-weeks-old Swiss Webster, Charles River) underwent surgery for implantation of a biofilm-coated stainless-steel ball within the muscle tissue of the left thigh. Female mice weighing 32–42 g (mean weight: 35 g) were anesthetized using isoflurane and oxygen (2–3 and 1% respectively). Subcutaneous injection of buprenorphine (slow release) was used to alleviate pain, along with carprofen delivered subcutaneously.

**Figure 1:**
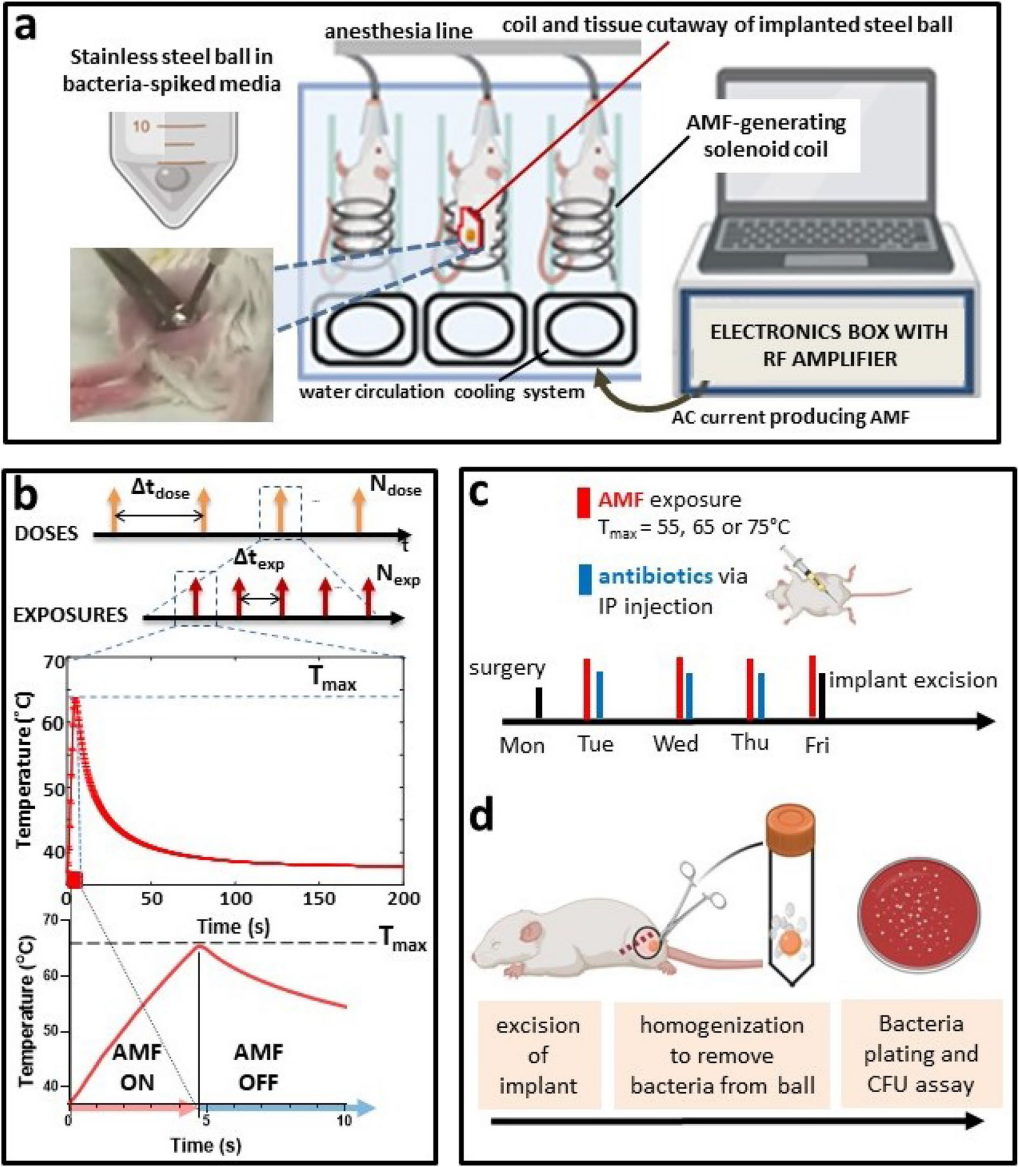
Workflow of AMF-antibiotic combination treatment. (a) Biofilm was grown on stainless steel balls submerged in bacteria-spiked media for 2 h and subsequently implanted in the thigh muscle of mice. Implant-bearing mice were then exposed to AMF within solenoid coils. (**b**) Parameter definition: AMF doses separated by hours (**Δt**_**dose**_), with each dose composed of multiple AMF exposures (N_exp_) delivered at intervals (**Δt**_**exp**_). Each exposure or pulse consists of AMF heating to a target temperature followed by temperature decline upon cessation of AMF. (**c**) The treatment regimen consisted of AMF exposure once a day up to 4 days with antibiotics administered post AMF via IP injection once a day for 3 days. (**d**) On day 4, 24 h after the 3rd antibiotic injection, stainless steel balls were excised, homogenized to remove bacteria and plated. Bacterial growth was quantified by colony forming unit (CFU) assay. Images created using BioRender.

Fur of the left thigh was shaved exposing the skin. The area was sterilized with povidone iodine and alcohol. An incision in the skin was then made at the muscle region to create a pocket to implant one stainless-steel ball (Fig. 1a). The incision was closed with coated VICRYL 5-0 absorbable sutures (Ethicon, Somerville, NJ). Implanted mice were allowed to recover for 24 h after surgery.

### iAMF treatment of mice

Implant-bearing mice were anesthetized using isoflurane (1–3%) and then exposed to AMF within custom solenoid coils (Fig. 1a). The mice were inserted into a solenoid coil, such that the implanted thigh was in the center of the coil. iAMF doses (N_dose_) composed of single or multiple AMF exposures were delivered 18 h after surgery at intervals (Δt_dose_) of either 12 or 24 h each day for 4 days (4–8 treatments) (Fig. 1b). AMF exposures consisted of an “AMF ON” period (t_exp_) to reach the target maximum temperature (T_max_ = 55, 65 or 75 °C) followed by an “AMF OFF” period (starting the instant AMF was shut off) for cool down to baseline (32–37 °C). These heating–cooling cycles were delivered at intervals of Δt_exp_ = 300 s. Mice treated with iAMF ramped up to T_max_ of 55 °C underwent N_exp_ = 24 exposure cycles lasting a total of 120 min per dose (where time of a single dose = N_exp_ × Δt_exp_), iAMF ramped to 65 °C underwent 12 cycles lasting 60 min per dose, and iAMF ramped to 75 °C underwent a single AMF cycle lasting 5 min in total per dose.

### Antibiotic treatment of mice

Antibiotic dose escalation studies were performed for each antibiotic to find a single daily dose resulting in a modest reduction (~ 0.5–1.0 log decrease) in CFU for the target pathogen. For S. *aureus*, 50 mg/kg ceftriaxone or 50 mg/kg rifampin was used, and for *P. aeruginosa*, 20 mg/kg ciprofloxacin was used. Eighteen hours after surgery, antibiotics were administered once a day via intraperitoneal (IP) injection for 3 consecutive days (regardless of whether iAMF was dosed once or twice daily).

### Combined AMF and antibiotic treatment of mice

Combined treatment was conducted 18 h after surgery with a dose of iAMF treatment as described, followed immediately afterwards by a dose of antibiotics (administered at room temperature). AMF and antibiotic treatments were given for their respective number of doses and dose intervals such that the last antibiotic treatment was given on the third day after surgery and the final AMF treatment was on day 4 followed immediately by animal euthanasia via CO2 inhalation (Fig. 1c).

### Sham treatment

Sham treatment animals were also implanted with a stainless-steel ball. Non-iAMF groups were kept in chambers maintaining the same environmental conditions as for the treated groups (exposure to isoflurane). Non-antibiotic groups received IP injections of nuclease-free water (diluent used to deliver antibiotics). Mice were euthanized at the equivalent time points as done in the active iAMF treatment groups.

### Assessment of biofilm elimination

On the fourth day of treatment, after AMF exposure, the mice were euthanized. The stainless-steel balls were explanted aseptically using narrow forceps, and the balls were placed in sterile tubes with 1 ml buffer (DPBS + 0.1% Triton) and beads. Balls were beaten for three minutes in intervals of 1 min in the bead beater and 1 min on ice. After vortexing, bacteria were plated on blood agar plates by drip plating^33^ and colony forming units (CFU) were quantified (Fig. 1d).

### Statistical analysis

All statistical analyses were performed using one-way ANOVA or t-test with Prism 9 (GraphPad, San Diego, CA). *p* values < 0.05 were considered statistically significant.

## Simulation of iAMF heating of metal ball implants and thermal dose calculation

**Figure 2:**
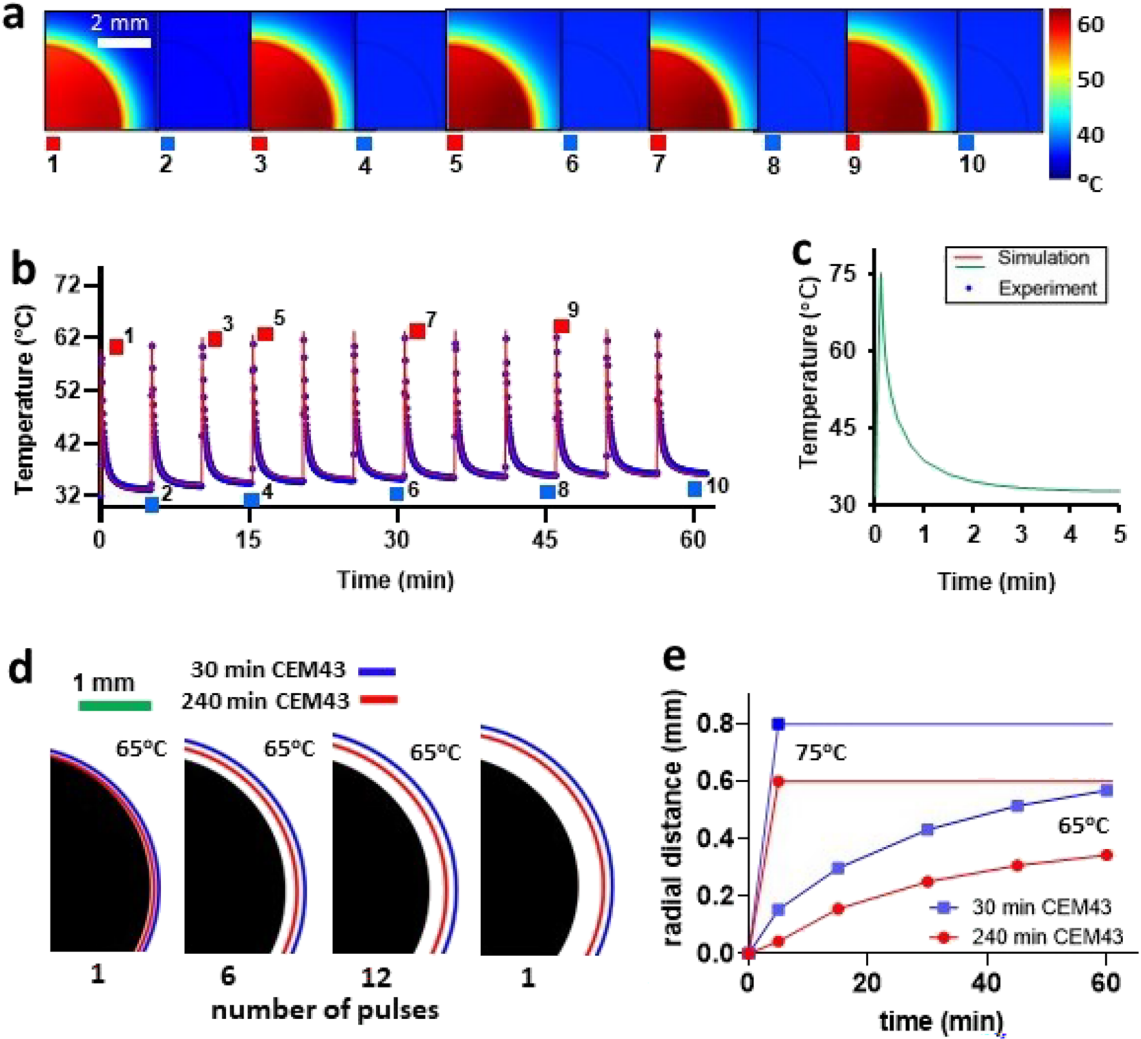
Simulation of intermittent alternating magnetic field (iAMF) heating of stainless-steel ball within muscle-mimicking medium and corresponding thermal dose calculations. (**a**) Simulation of temperature distribution through cross-section of steel ball and muscle tissue-mimicking gel at various temperature maxima and minima (red and blue numbered squares) during 12 cycles of iAMF heating to a 65 °C target. (**b**) Graph of temperature vs. time during heating and cooldown cycles (measured and simulated) at a point within the center of the metal ball corresponding to simulations in panel a. (**c**) Simulation of 1 AMF cycle to 75 °C. (**d**) Thermal dose boundaries for reversible (30 min CEM43, blue) and irreversible (240 min CEM43, red) damage to muscle tissue after 1, 6 or 12 heating cycles to 65 °C or 1 cycle to 75 °C. (**e**) Radial expansion of thermal dose boundary from ball surface for reversible (blue) and irreversible (red) damage to muscle tissue vs. exposure time to iAMF heating for 12 cycles to 65 °C or 1 cycle to 75 °C.

Finite element simulations of the heating of a metal ball suspended in tissue-like medium exposed to iAMF with parameters corresponding to the treatments in this study were performed using the commercial simulation software COMSOL Multiphysics (Comsol v5.5, Los Angeles, CA, USA). A quasi-static approximation of Maxwell’s equation and Penne’s bioheat transfer model was applied for the electromagnetic and thermal simulations. The 3D physical model of the metal ball at various time points as it cycles through AMF pulses in tissue-like medium is shown in Fig. 2a. Simulations made use of free tetrahedral meshing with boundary layers to capture the skin effect. Grid independent studies were conducted and an optimum number of 853,944 elements were used for analysis.

Physical properties of the simulated ball (316L stainless-steel) include a density of 8000 kg/m^3^, electrical conductivity of 1.351 × 10^6^ S/m, relative permittivity of 1, thermal conductivity of 16.3 W/(m K) and a specific heat of 500 J/(kg K). Corresponding properties of the tissue-like medium were 1090 kg/m^3^, 0.384 S/m, relative permittivity of 6377, thermal conductivity of 0.49 W/(m K) and 3421 J/(kg K), and blood perfusion of 3.6 kg/(m^3^ s^1^)^34^.

Further calculations were performed in COMSOL to estimate the thermal dose^35^ to tissue immediately surrounding the implant. Thermal dose can be expressed in terms of cumulative equivalent minutes of exposure at the reference temperature of 43 °C (CEM43). Based on the normalization of measurements of a variety of tissues exposed to different temperatures for various lengths of time, equivalent time of exposure thresholds for tissue damage at 43 °C have been determined^36^. While less than 30 min of exposure at 43 °C (30 CEM43) causes no damage to muscle tissue, greater than 240 min (240 CEM43) causes irreversible damage, with reversible damage occurring between these values^37^.

### Ball heating experiment and validation of simulation

To validate heating and CEM43 simulation, a steel ball identical to the ones transplanted was heated with AMF to 65 °C, then allowed to cool at 5 min intervals for a total of 12 pulses over 60 min, following the treatment protocol as used in mice (Fig. 2). The animal was positioned such that the ball was at the center of the coil.

### Histopathology for evaluation of thermal damage to tissue

The tissue surrounding the implanted steel ball was processed for histopathological analysis to evaluate thermal damage associated with AMF treatment. Mice were either euthanized immediately after the fourth AMF treatment or survived an additional 28 days to observe any delayed effects of AMF on tissue. The leg was harvested and fixed in formalin for 7 days. The tissue was then transferred into a decalcifying solution (Thermo Fisher Scientific, Waltham, MA) for 14 days allowing the tibia and femur to decalcify. When the decalcification was complete, the ball was carefully removed from the leg, and the limb was embedded in a paraffin block. Hematoxylin & eosin (H&E) stained tissue sections were acquired from the region where the ball had been present. These sections were then examined by a pathologist in a blinded fashion to evaluate the outward extent of thermal damage around the implant.

## Results

### Simulation of temperature distribution and thermal dose to implant and surrounding tissue

The intermittent AMF heating of a steel ball immersed in a medium mimicking the properties of muscle tissue was numerically simulated to model the dynamics of heat distribution within the implant and surrounding tissue (Fig. 2). Simulations were representative of typical iAMF doses (either 12 cycles of heating to 65 °C delivered at 5 min intervals or one cycle to 75 °C) utilized in this study. As observed in the simulated cross-section through the steel ball and tissue medium (Fig. 2a), at a peak implant temperature of ~ 65 °C, significant temperature increases in the surrounding tissue-like medium stayed well within 1 mm of the ball, even after multiple exposures. In the cooldown phase of each cycle, both the steel ball and surrounding medium returned to the baseline temperature (~ 32 °C) with minimal temperature accumulation over the course of the 60 min dose (Fig. 2b). To check the consistency of the simulations against in vivo iAMF experiments, the temperature of a steel ball surgically implanted in the leg muscle of a mouse was measured utilizing a sensor embedded within the ball. As shown in Fig. 2b, the measurements (blue dots) overlapped with the simulation curve (red line) with minimal variation. Fitting rise and decay portions of the simulated and measured temperature data to exponential growth and decay curves, R^2^ goodness of fit was greater than 0.97 in all cases. For comparison, the single pulse 75 °C AMF simulation is shown in Fig. 2c.

From the simulation of dynamic iAMF temperature distribution, we calculated the accumulated thermal dose within the tissue volume at radial distances from the implant surface into the tissue based on the CEM43 model (Fig. 2d–e). As iAMF heating time (or AMF pulse number) increased, the boundaries of 240 CEM43 (irreversible tissue damage) and 30 CEM43 (completely reversible tissue damage) increased. However, even after 12 AMF cycles, 240 and 30 CEM43 were only 0.57 mm and 0.35 mm from the metal implant surface, respectively, where all tissue beyond these boundaries would have no damage.

### iAMF and antibiotics are effective at reducing biofilm in vivo

**Figure 3:**
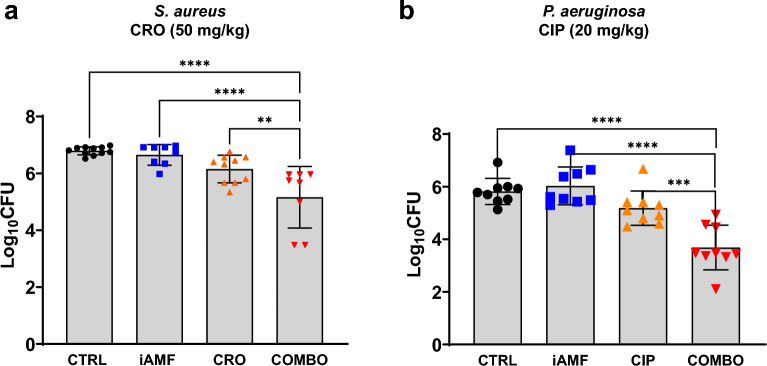
Bacterial log reduction of biofilm of (**a**) Gram-positive (S. *aureus*) with ceftriaxone [CRO] at (50 mg/kg) or (**b**) Gram-negative (*P. aeruginosa*) with ciprofloxacin [CIP] at (20mg/kg) after a single cycle of iAMF heating per day (T_max_ = 75 °C; N = 8), antibiotics (ABX; N = 10) or iAMF∙ABX combined therapy (iAMF∙ABX; N = 10) versus sham (no antibiotics or iAMF; N = 11). Error bars indicate SD. Results were significant at *p* = 0.0061 (**), 0.0004 (***) and < 0.0001 (****) for specific comparisons shown using one-way ANOVA with Tukey’s multiple comparisons test. The average log_10_CFU initial bacterial count on the unimplanted ball for *S. aureus* and *P. aeruginosa* was 5.0 and 4.6, respectively. Experiments were repeated three times.

Having modeled and characterized the heating effects of iAMF, in vivo experiments were conducted to compare its effects alone and in combination with antibiotics on different strains of biofilm-infected implants. One stainless-steel ball, with either *Staphylococcu*s *aureus* or *Pseudomonas aeruginosa* biofilm, was implanted in the leg muscle of mice and underwent four consecutive days of treatment with either sham (no iAMF, no antibiotics), one pulse of 75 °C iAMF, antibiotics alone or iAMF-antibiotic combination, after which remaining biofilm CFU was determined from extracted implants (Fig. 3). *S. aureus* infected mice were treated with sham iAMF, iAMF alone, ceftriaxone (50 mg/kg), or combination treatment while *P. aeruginosa* infected groups were treated with sham iAMF, iAMF alone, ciprofloxacin (20 mg/kg) or combination treatment.

After 4 days of therapy, iAMF-antibiotic combination treatment resulted in reduction of 1.63 log of *S. aureus* biofilm and a 2.13 log reduction in *P. aeruginosa* biofilm compared to sham treatment (*p* < 0.0001). In contrast, compared to sham treatment, neither iAMF nor antibiotic monotherapies had a significant effect on biofilm CFU for either strain. Compared to iAMF monotherapy, combination treatment further reduced *S. aureus* and *P. aeruginosa* biofilm CFU by 1.49 log and 2.34 log respectively (*p* < 0.0001). Compared directly to antibiotic monotherapy, combination treatment further reduced *S. aureus* and *P. aeruginosa* biofilm CFU by 1.00 log (*p* = 0.0061) and 1.50 log (*p* = 0.0004) respectively.

### Effect of iAMF target temperature on biofilm CFU reduction

**Figure 4:**
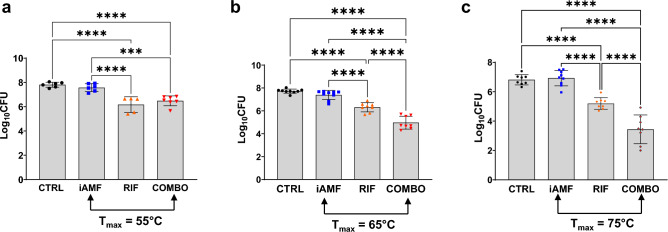
Bacterial log reduction of S. *aureus* biofilm after iAMF heating to various temperatures (T_max_; N = 6, 9, 9 respectively), rifampin administration (50 mg/kg, RIF; N = 5, 8, 8 respectively) or iAMF∙RIF combined therapy (iAMF∙RIF; N = 7, 8, 8 respectively) versus sham (no ABX or iAMF; N = 6, 9, 8 respectively) treatment. In iAMF-treated animals, implants were heated to T_max_ of (**a**) 55 °C (**b**) 65 °C, and (**c**) one cycle 75 °C per day. Error bars indicate SD. Results significant at *p* = 0.0007 (***) and < 0.0001 (****) for comparisons shown using one-way ANOVA with Tukey’s multiple comparisons test. Experiments in (**a**) were repeated twice and in (**b**, **c**) were repeated three times.

Having observed bactericidal effects with single pulses of AMF at a target of 75 °C, treatment efficacy was investigated at lower temperatures. To compensate for temperature reduction, 12 pulses of AMF were applied for a peak temperature target of 65 °C, while 24 pulses were applied for a peak temperature target of 55 °C (Fig. 4). To explore the impact of other antibiotics when combined with iAMF, rifampin (50 mg/kg) was investigated for activity in *S. aureus* biofilm.

Combination treatment again demonstrated enhanced bactericidal results compared to the effects of either monotherapy alone. At iAMF treatment of 65 °C and 75 °C, iAMF-rifampin combination treatment further reduced S. *aureus* biofilm CFU by 1.35 and 1.76 log compared to rifampin alone (*p* < 0.0001). Combination treatment at 55 °C, however, demonstrated no significant benefit over antibiotic monotherapy. Compared to iAMF heating alone, combination treatment resulted in significantly greater reduction of *S. aureus* biofilm CFU at all target temperatures, with comparative log reductions of 2.43 (65 °C; *p* < 0.0001), 3.48 (75 °C; *p* < 0.0001) and 1.09 (55 °C; *p* = 0.0007). Compared to sham treatment, combination therapy yielded 1.33, 2.76 and 3.37 log reductions in CFU at 55 °C, 65 °C and 75 °C, respectively (*p* < 0.0001). Comparing monotherapies, rifampin was more effective at reducing biofilm CFU than iAMF alone at all temperatures tested (*p* < 0.0001) with log reductions 1.40, 1.07 and 1.72 more than iAMF at 55 °C, 65 °C and 75 °C, respectively.

### Effect of iAMF dosing frequency on biofilm CFU reduction

**Figure 5:**
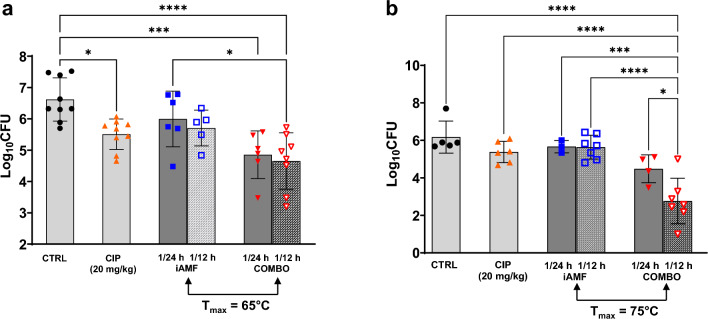
Bacterial log reduction of PAO1 P. *aeruginosa* biofilm on implant after AMF treatment once per 24 h or once per 12 h in combination with ciprofloxacin [CIP] at 20 mg/kg (iAMF∙CIP), after 20 mg/kg ciprofloxacin alone (CIP) or after iAMF alone at implant T_max_ of (**a**) 65 °C, and (**b**) one cycle of 75 °C per dose. Error bars indicate SD. Results significant at *p* = 0.0175–0.0287 (*), 0.0006 (***, panel a), 0.0003 (***, panel b) and < 0.0001 (****) for comparisons shown using one-way ANOVA with Tukey's multiple comparisons test. Experiments in (**a**) were repeated three times (N = 5–9) and (**b**) were repeated twice (N = 3–7).

In addition to modulating target temperature and pulse number, the effects of altering iAMF dosing frequency were investigated. Mice with *P. aeruginosa* biofilm infected implants were exposed to iAMF dosing once every 24 h or once every 12 h for four days at 65 °C (12 pulses) or 75 °C (1 pulse) alone, in combination with ciprofloxacin (20 mg/kg) or treated with ciprofloxacin alone (Fig. 5). Just as is seen with antibiotics, iAMF showed dose-dependent effects with increasing number of iAMF treatments resulting in further CFU reduction when combined with antibiotics. This effect was particularly pronounced at a target temperature of 75 °C where a once every 12 h dosing strategy resulted in a 3.41 log reduction compared to sham treatment (*p* < 0.0001). However, iAMF monotherapy did not result in significantly greater CFU reductions when given twice versus once a day.

### Histopathologic analysis of tissue surrounding iAMF-treated metal ball implants

**Figure 6:**
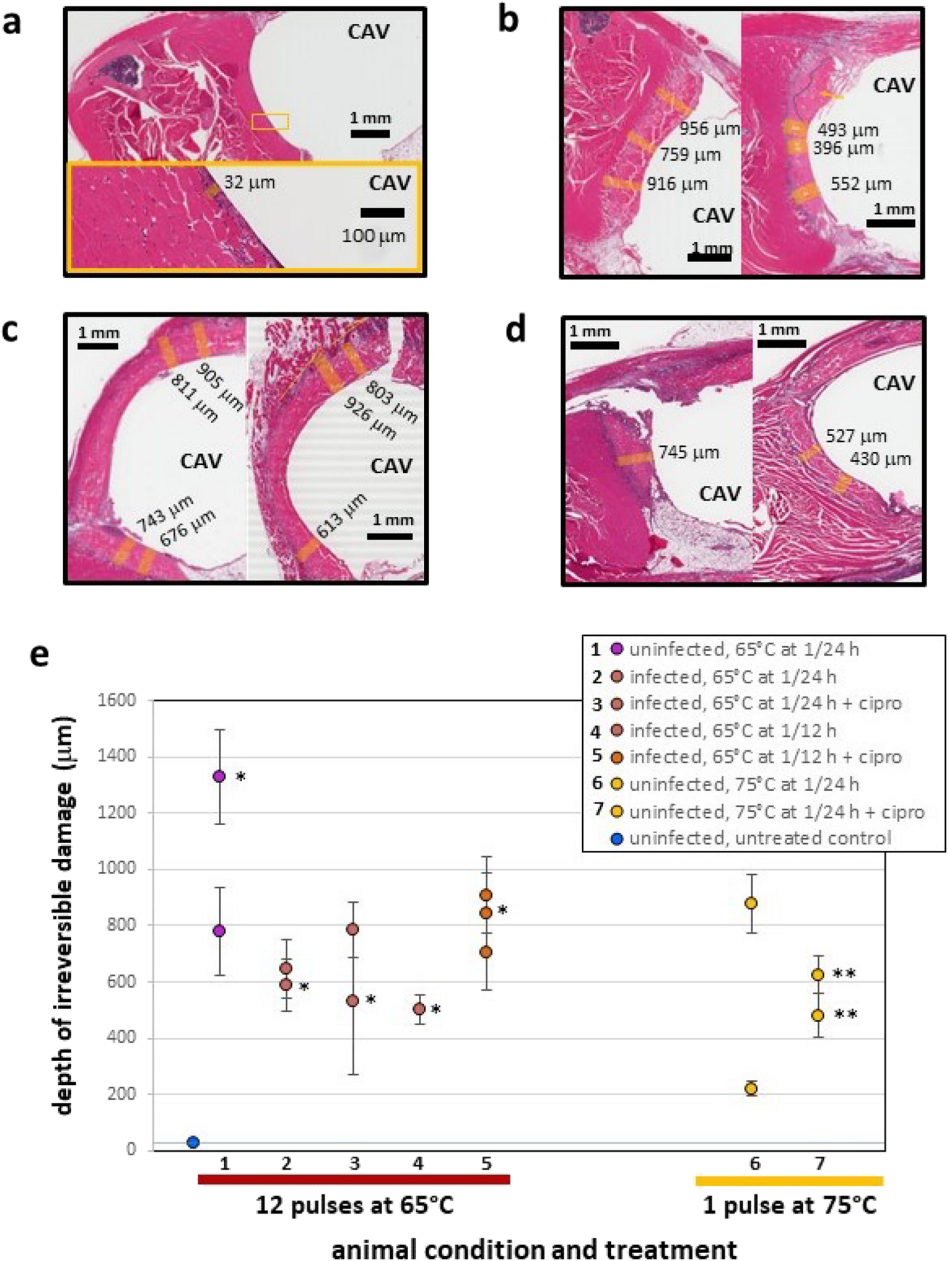
Histopathology of muscle tissue in contact with iAMF-heated steel ball implants from infected and uninfected iAMF treated mice with/without antibiotics. Representative tissue sections from animals treated 4 consecutive days under various conditions. (**a**) Sham-treated uninfected mouse with minimal myofiber necrosis and attendant inflammatory reaction at implant site. Inflammatory cells, likely neutrophils, extend ~ 20–30 mm into tissue from edge of implant cavity (CAV). (**b**) Uninfected mice treated with iAMF (T_max_ = 75 °C) without (left) or with (right) ciprofloxacin. (**c**) iAMF-treated (T_max_ = 65 °C) mouse infected with *P. aeruginosa* and treated with ciprofloxacin (20 mg/kg, left) and uninfected mouse (right). (**d**) *P. aeruginosa* infected mice dosed with iAMF (T_max_ = 65 °C) every 12 h for 1 day (left) and 4 days (right). Measurements of irreversible tissue damage (myofiber necrosis) depth from edge of CAV indicated by orange bars. (**e**) Depth of irreversible damage into tissue versus mouse condition and treatment. Numbered condition and treatment of each animal listed in legend. Error bars indicate SD of measurements of individual animals. *Treated 1 day and euthanized. **Treated 7 consecutive days and euthanized.

To assess the extent of tissue damage under different conditions, histopathologic analysis of the implant-tissue interface was performed. Tissue of sham-treated animals (no iAMF, infection or antibiotics administered) in contact with the steel ball implant for four days exhibited negligible myofiber necrosis and infiltration of inflammatory cells at a depth of ~ 20–30 μm from the edge of the implant-tissue interface (Fig. 6a). For the treated animals, histology was analyzed on tissues of animals under 7 combinations of conditions (infected or uninfected; iAMF treatment at the 65 °C or 75 °C target temperature). These treatment combinations (Fig. 6e) were applied for 4 consecutive days except where noted.

Representative tissue samples (Fig. 6b treated at 75 °C, Fig. 6c at 65 °C, and Fig. 6d at 65 °C at twice the frequency and total dose as in **c**) illustrate myofiber necrosis (irreversible damage) and inflammation surrounding the implant site at varying degrees of severity and depths into the tissue. Measurements were taken at various locations along the periphery of the implant-tissue interface perpendicular to the cavity edge in regions free of artifact due to surgical procedures or tissue processing. Such regions were mainly located on the side proximal to the femur or opposite to it and were selected as representative of tissue damage due specifically to AMF-heating. All animals were euthanized immediately after completion of treatment. Comparing uninfected mice treated only with iAMF (Fig. 6b, left, at 75 °C and Fig. 6c, right, at 65 °C), measurements of tissue damage treated at 75 °C ranged from 759 to 956 μm from the implant, while measurements of tissue damage treated at 65 °C, ranged from 613 to 926 μm, with no significant difference between the two sets of measurements.

In total, depth measurements of necrosis and inflammation were obtained for 14 mice where histology sections were viable for analysis (Fig. 6e). A total of 48 measurements taken revealed a mean and standard deviation of 690 ± 268 μm of tissue damage across all conditions. No significant differences in the mean depth of tissue damage were observed based on peak temperature or frequency of dosing. For animals treated with iAMF at 65 °C once per day, mean depth of tissue damage was 745 ± 297 μm (from n = 22 total measurements of 6 mice) while for animals treated at 65 °C twice per day, mean depth of damage was 721 ± 193 μm (from n = 14 measurements of 4 mice).

## Discussion

Infections of metallic implants cause significant morbidity and mortality. For prosthetic joint infections (PJI), removal of the implant is frequently needed for adequate treatment. This is largely due to the formation of biofilm on the implant surface, rendering antibiotics ineffective. We have previously demonstrated that AMF can reduce biofilm in a temperature and time-dependent fashion^29,30^. While AMF offers several distinct advantages in its clinical implementation over other heat-generating technologies aimed at eliminating biofilm from implant surfaces, it has yet to demonstrate full safety and feasibility in vivo. Here in our first in vivo proof of concept efficacy study, we show that iAMF when combined with antibiotics results in modest decreases in biofilm compared to either sham (no treatment) control, iAMF or antibiotic treatment alone. Importantly, this effect was seen in both Gram-negative and Gram-positive pathogens and multiple antibiotics. We included rifampin alone for these initial studies to understand whether there are synergistic effects with AMF and individual antibiotics. However, in practice this antibiotic is utilized in combination with other agents. Although the in vivo differences were not as significant as those we observed in vitro^30^, it is clear that AMF can be delivered to animals. The time–temperature-antibiotic dosing parameters were limited in this study and future studies will help further optimize the treatment effects that were seen. Demonstrating that AMF could be used in multiple pathogen-antibiotic settings is critical as the ultimate clinical use of this technology will be for biofilm-related infections that are agnostic of the pathogen causing them. While the final parameters of iAMF that will be used clinically remain to be determined, this study revealed useful parameters that will be utilized in future large animal studies. Although AMF by itself has the ability to eradicate biofilm^29^, intermittent AMF (iAMF) is designed to strike a balance between providing efficacy when combined with antibiotics and limiting damage to surrounding tissue. The synergistic effects of combining iAMF with antibiotics in the in vitro setting was recently shown by our group^30^. It was not clear that these effects would translate in vivo, but importantly, they do. Furthermore, these effects were evident despite the brief period of time that iAMF was used for. Depending on the peak temperature and number of pulses used, total “doses” of iAMF ranged from 5 min per dose per day (75 °C, one pulse) to 60 min per dose per day (65 °C). The number of treatments for iAMF alone did not produce a bactericidal effect; reduction of biofilm was seen only with iAMF in combination with antibiotics. Increasing the number of iAMF-antibiotic treatments at 65 °C (Fig. 5A) was not statistically significant. However, comparison of iAMF-antibiotic dosing once every 12 h versus once every 24 h did show further reduction at 75 °C illustrating the importance of both temperature and number of AMF treatments in reducing biofilm burden.

These studies demonstrated that, at least for *S. aureus* and rifampin, there was a lower limit of temperature (55 °C) that did not seem to provide additional reduction in biofilm over rifampin alone. The lower limits of temperature and time with iAMF that could still provide efficacy are being explored across multiple pathogens and antibiotics in ongoing studies. In addition, higher temperatures (75 °C) demonstrated greater biofilm reduction compared to 65 °C. Thermal dose received by the surrounding environment (both tissue and biofilm) varies with depth from the implant surface. Based on our simulations (Fig. 2e), the total thermal dose received at a depth of ~ 600 μm from the 75 °C single pulse (240 CEM43) was predicted to be 8 times the total dose received from 12 pulses at 65 °C (30 CEM43). Thus, total thermal dose may play a significant role in the eradication of biofilm in iAMF-antibiotic combination treatment. Other important findings of this study included that increased number of doses of iAMF resulted in a greater reduction of biofilm, at least for certain pathogen-temperature-antibiotic combinations, particularly for *P. aeruginosa* and ciprofloxacin with an iAMF T_max_ of 75 °C. Multiple dosing parameters of iAMF are being explored for other pathogens and antibiotics to help define the most efficacious treatment algorithm.

Important for the eventual deployment of iAMF is maintaining efficacy while limiting toxicity. While safety will be further assessed in formal ongoing and future safety studies, we examined the extent of tissue damage surrounding the implant under different treatment conditions. Histopathological analysis was performed by a pathologist and revealed that iAMF resulted in a small boundary of visually apparent thermal damage that was < 1 mm in depth. The use of intermittent pulsing of AMF that allows for a brief exposure at a particular temperature followed by a cooling down period allows for an anti-biofilm effect while maintaining limited thermal necrosis. Ideally, based on the CEM43 standard, the thermal dose that tissues are exposed to can be calculated from measured or simulated temperature distributions and equated to the extent of tissue damage^33,34^. A steel ball was chosen as the metallic implant because it is perfectly symmetrical and therefore simulation models were fairly easy to create. In addition, damage to tissue was fairly evenly distributed around the implant cavity. While the model predicts both reversible (30 min) and irreversible (240 min) CEM43 boundaries, actual reversible damage to tissues is difficult to identify. Hence, considering both model simplicity and experimental complexity in this study, we compared the measured irreversible damage depth with the 240 min accumulation prediction. While for 75 °C iAMF, the mean depth of irreversible damage was very close to the predicted, the 65 °C iAMF mean depth was twice that predicted however, further histopathologic analysis will be needed as the number of mice analyzed in the uninfected state was small. Limitations of this study included minor variations in positioning mice in the iAMF treatment coils. Orientation and shape of the implant relative to the magnetic field is an important determinant of eddy currents and spatial heating distribution on the implant. As the goal was to have the steel ball at the center of the coil for duration of treatment, any deviation in position from the center would result in a reduced magnitude of the magnetic field. Furthermore, the ability of the ball to drift slightly from its original position in an animal over the course of several days was possible, although whether the ball had moved was apparent upon dissection and was excluded from the histological analysis component of the study. Importantly, this in vivo model used a perfectly symmetrical implant and does not represent more complex shaped implants. The choice of a spherical ball in this study was used to eliminate the impact of orientation as a confounding variable. It is likely that more complex shapes will require different treatment algorithms, which we are exploring in ongoing studies.

Another important consideration in our study is the composition of the model stainless steel implant. Multiple alloys (such as cobalt-chrome and titanium) are often used in joint implants. These materials can have different electrical properties and heat at different rates in a magnetic field. From our in vitro investigations and unpublished work in large animals, we have not observed any statistically significant difference in biofilm reduction across metal alloys. Therefore, we conclude that, at the reported field strengths, the predominant mechanism leading to the enhanced efficiency of antibiotics is a direct heating effect.

This study illustrates the effects of iAMF in combination with antibiotics in a small animal model, the first of its kind. We discovered that implantation of the stainless-steel ball presented a few challenges: the ball could not be implanted next to or in the bone due to its relative size and risk of impinging on critical structures such as the sciatic nerve. Further, incubation times of bacteria on the ball before implantation were necessarily short as longer incubations resulting in dissemination of the infection. Thus, this model involves an early biofilm in a non-osseus environment. Nevertheless, the small animal model aids in confirming that the relationship between iAMF and antibiotic combined treatment recapitulates our in vitro findings. To study the applicability of safety and effectiveness of the exposure to orthopedic implants, continuing studies are currently focusing on bone implants in large animal models with a longer duration of infection (21 days). Despite these limitations, this study echoes our findings in vitro, specifically, that iAMF in combination with antibiotics reduced biofilm to a greater extent than either treatment alone. This effect caused minimal surrounding tissue damage and the treatment was effective in both prototypic Gram-positive and Gram-negative pathogens. Future studies will explore the mechanism of this iAMF-antibiotic interaction as well as assess safety in a large animal implant model. In the future, iAMF could play an important role in treating biofilm infections of metallic implants.

## Data Availability

The datasets used and/or analyzed during the current study are available from the corresponding author on reasonable request.
